# Supplementary material for: A reversible dendrite-free high-areal-capacity lithium metal electrode
Source: Nat Commun. 2017 Apr 25;8:15106. doi: 10.1038/ncomms15106 (PMC5414052; doi:10.1038/ncomms15106)
Supplement: Supplementary Information — Supplementary Figures [file ncomms15106-s1.pdf]

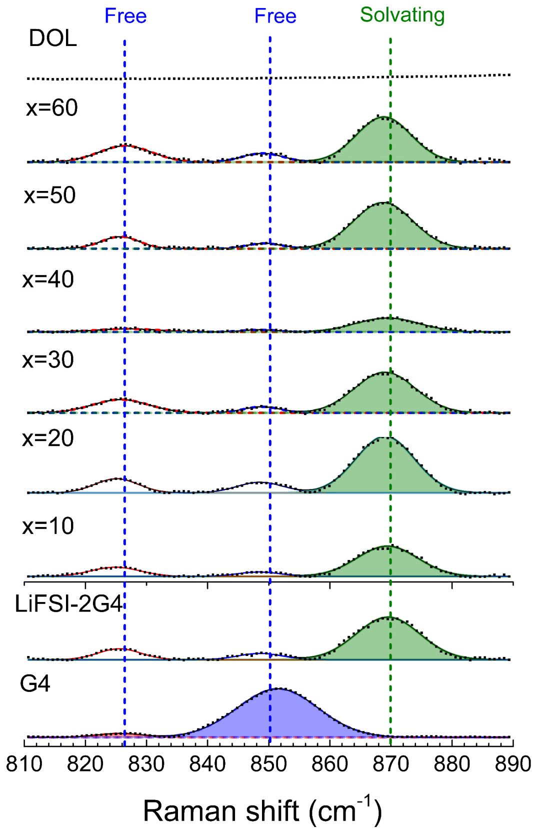

Supplementary Figure 1. Raman spectra for LiFSI-2G4-x vol% DOL in the frequency ranges of 810–890 cm^{-1} at 298 K.

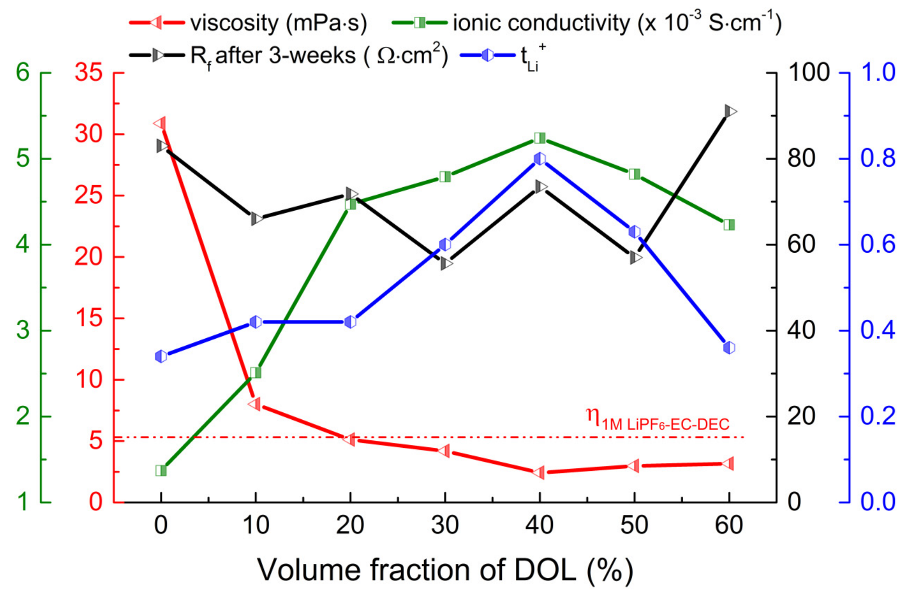

Supplementary Figure 2. Physiochemical and electrochemical properties of LiFSI-2G4- x vol% DOL at 25 ºC.

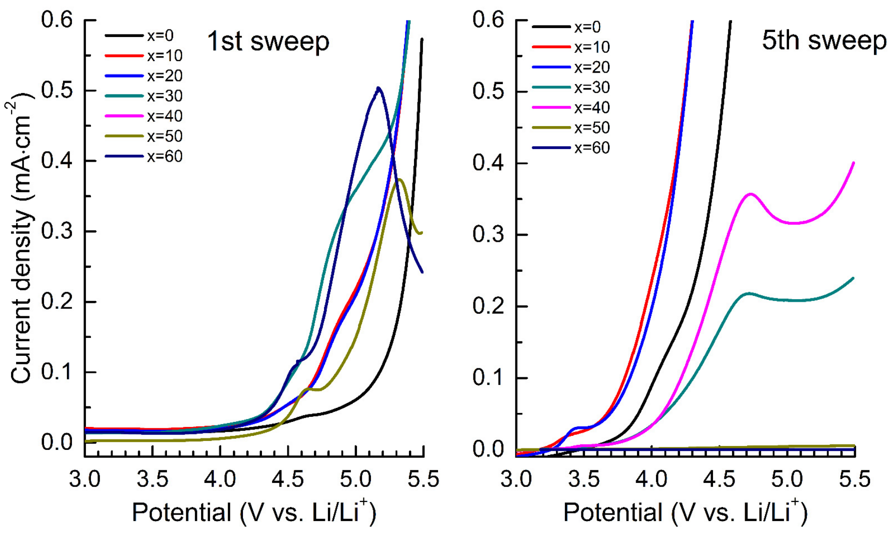

Supplementary Figure 3. Electrochemical stability of LiFSI-2G4-x vol% DOL using CV sweeps in the coin cell of Li|stainless steel electrode.

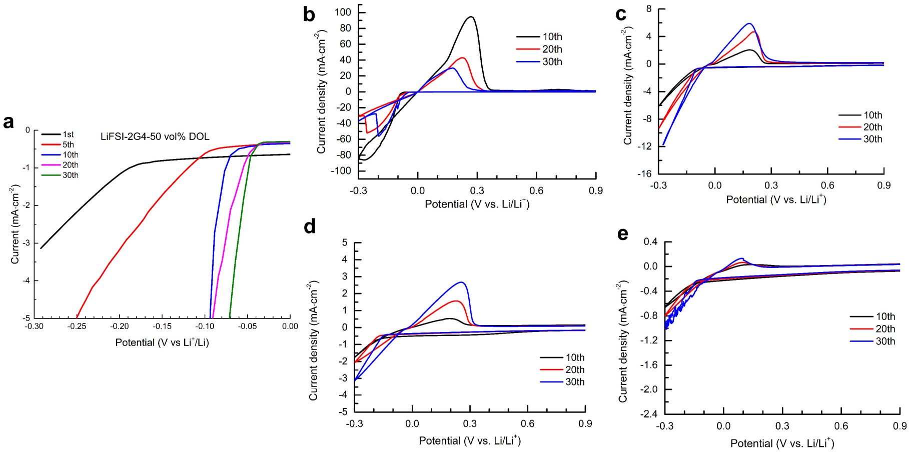

Supplementary Figure 4. Cyclic voltammograms of Li deposition/stripping in different electrolytes using Cu as the working electrode and Li as the reference and counter electrode. The scan rate was 50 mV s^{-1}. (a) the deposition behavior of lithium in LiFSI-2G4-50 vol% DOL at different cycles. (b) LiFSI-2G4-50 vol% DOL; (c) LiFSI-2G4; (d) LiFSI-G4; (e) LiFSI-2G4-50 vol% G4.

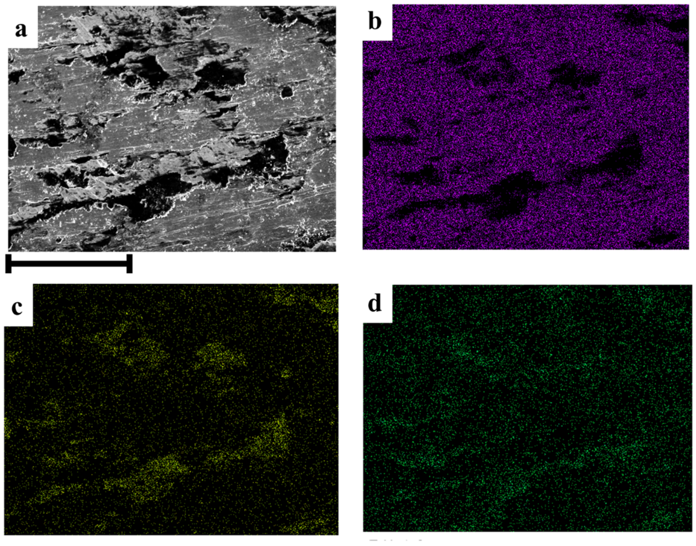

Supplementary Figure 5. EDX elemental mapping of Cu electrode after CV-premodulation. (a) Cu electrode (b) Cu-element mapping (c) O-element mapping (d) F-element mapping. Scale bar: 50 μm.

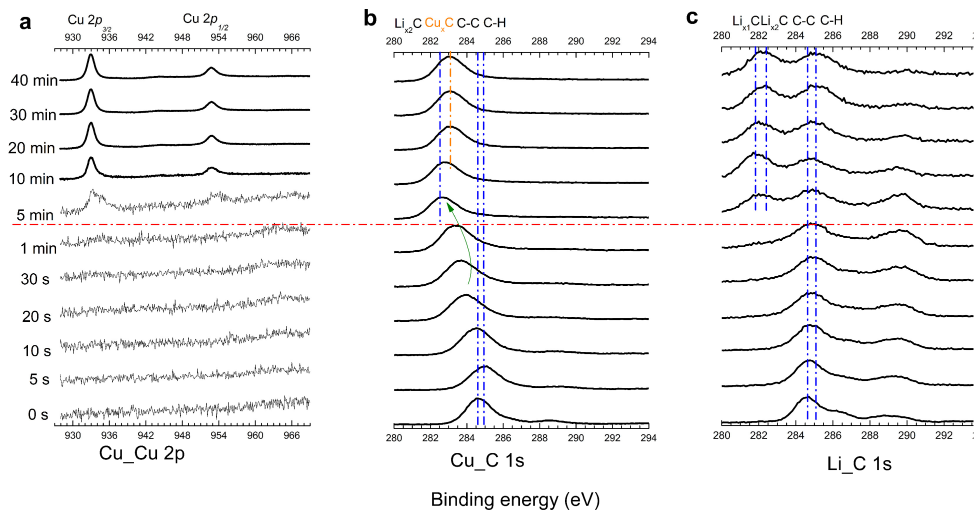

Supplementary Figure 6. A new SEI film chemistry formed onto Cu and native SEI of Li substrate in the LiFSI-2G4-50 vol% DOL by CV-premodulation.

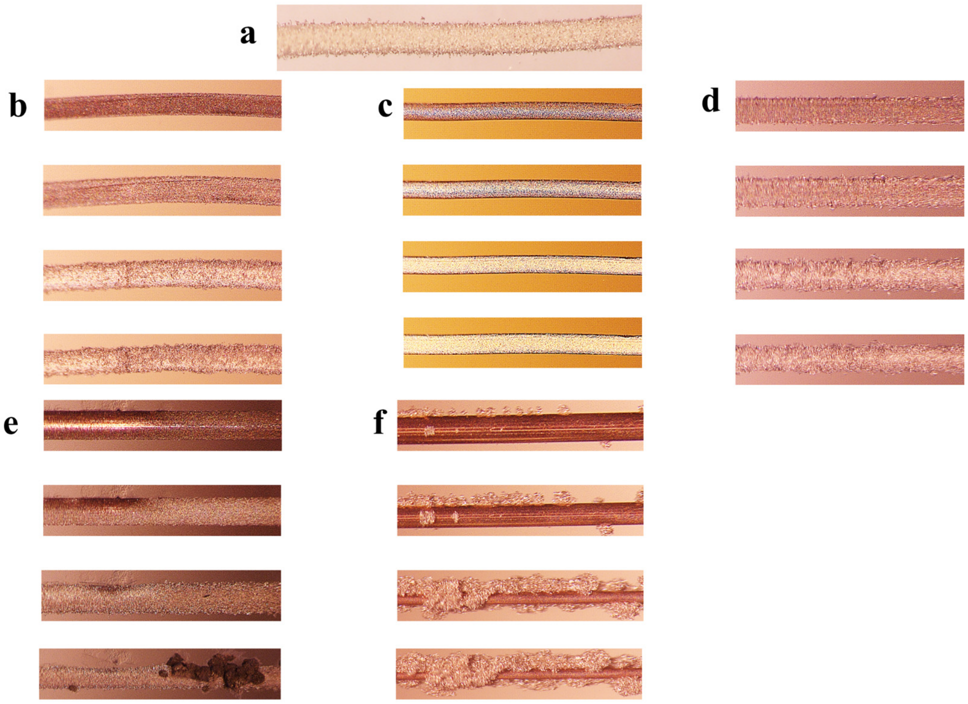

Supplementary Figure 7. Morphology evolution of electrodeposited lithium with time on the Cu electrode in different electrolytes. (a) the electrodeposited Li from Fig. 1e at 120 min. (b-e) deposition-time dependence (0.5 min, 2 min, 12 min, 18 min) of morphology evolution of lithium electrodeposits at 5 mA cm^{-2} and ambient temperature after CV-premodulation in the LiFSI-2G4-50 vol% G4 (b), LiFSI-2G4-50 vol% DOL (c), LiFSI-2G4 (d), LiTFSI-2G4 (e) and $LiPF_6$-EC-DEC (f), respectively.

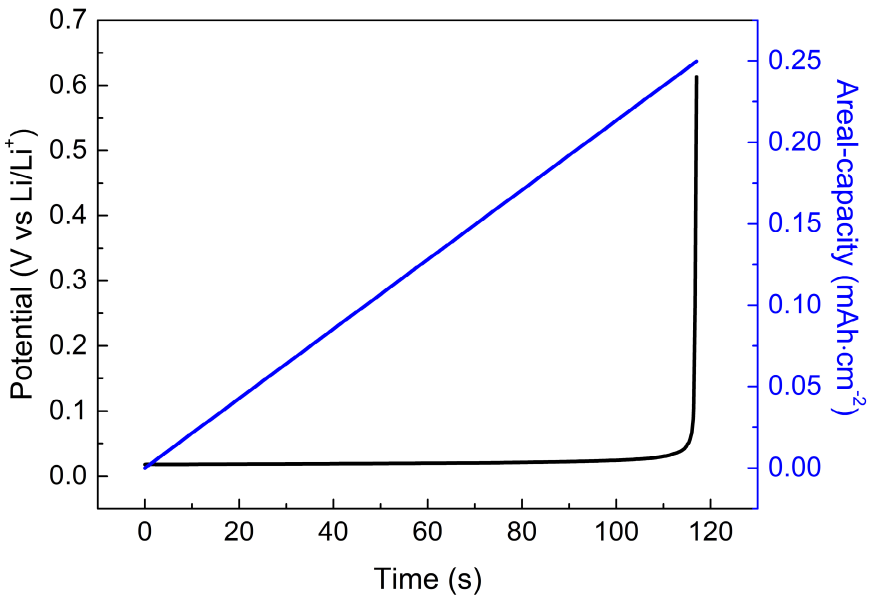

Supplementary Figure 8. Determination of Q_r by stripping Li from the Cu electrode of the Li|Cu cell after CV-premodulation at 5.0 mA cm^{-2}.

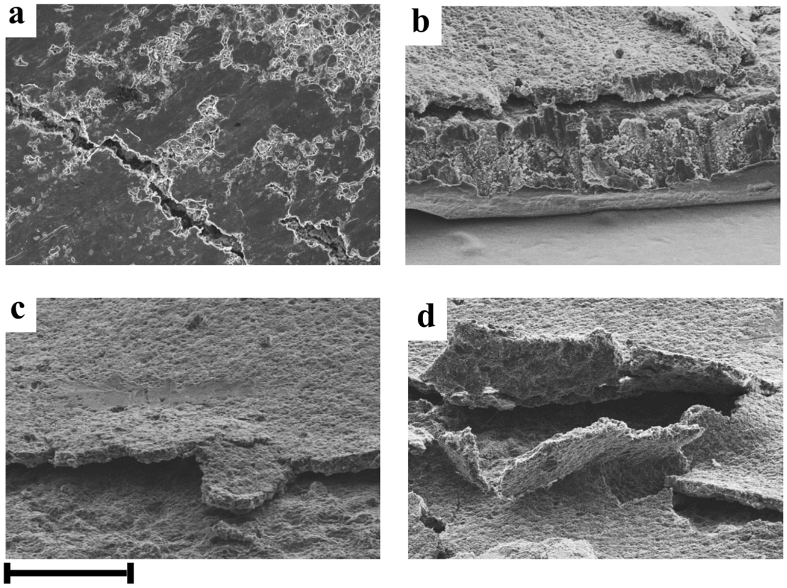

Supplementary Figure 9 Morphology of the lithium electrode after 100 cycles at 5.0 mA cm^{-2} (top-surface view, cross-section view, inclined-plane view). Each figure has a different amplification. (a) scale bar: 100 μm; (b-c) scale bar: 200 μm; (d) scale bar: 300 μm.

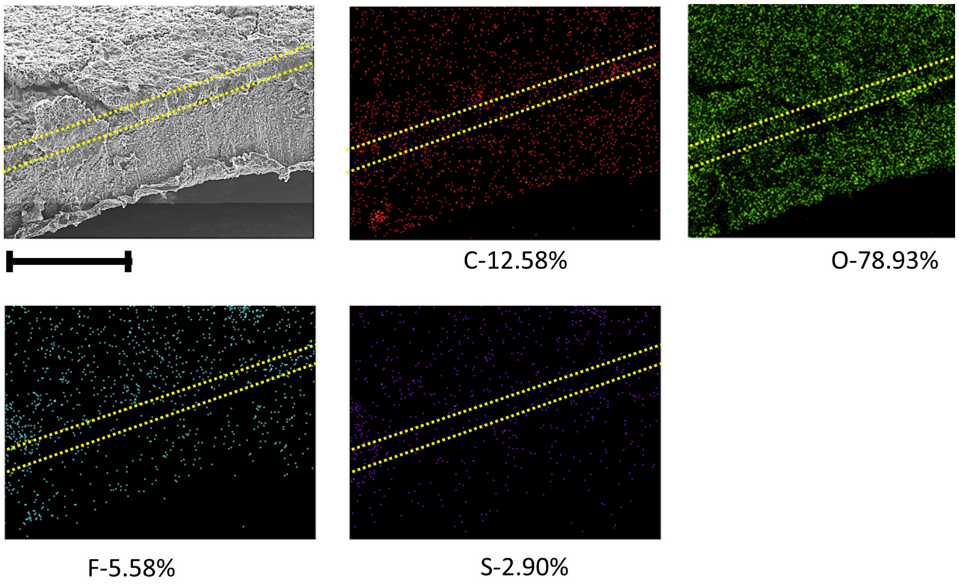

Supplementary Figure 10. EDX elemental mapping of the cross-sectional lithium electrode. The atomic percent is shown below the corresponding figure. Scale bar: 100 μm.

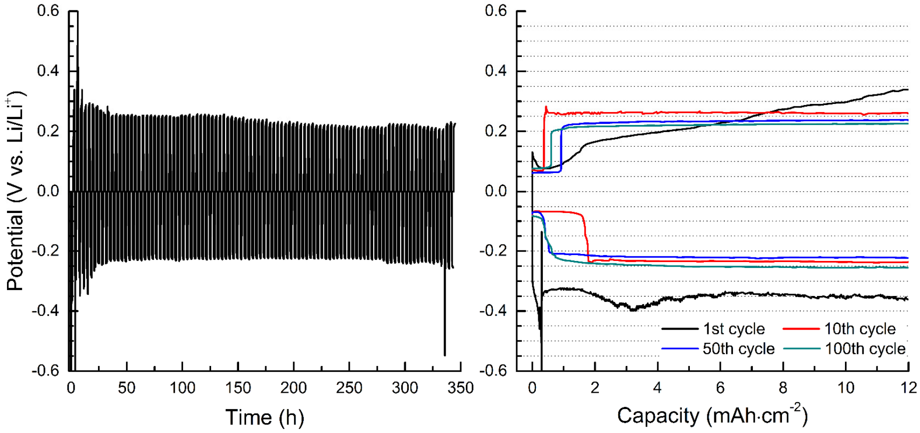

Supplementary Figure 11. Cyclic performance of a Li|Li cell at 10.0 mA cm^{-2} using LiFSI-2G4-50 vol% DOL. The delivered areal-capacity for each cycle was fixed at 12 mAh cm^{-2}. The right plot on the individual cycle is derived from the left plot.

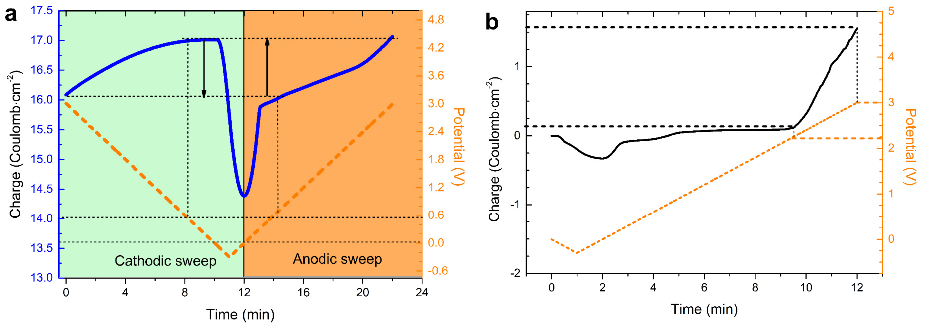

Supplementary Figure 12. Curves of charge vs. time for Li|Cu cells. (a) the 30th-sweep of a Li|Cu cell at 5.0 mV s^{-1} and 25 ^{o}C in the range of -0.3-3.0 V. (b) the first-sweep of a Li|Cu cell at 5.0 mV s^{-1} and 25 ^{o}C in the range of -0.3-3.0 V after five pre-sweeps at 5.0 mV s^{-1} and 25 ^{o}C in the range of -0.3-0.9 V.

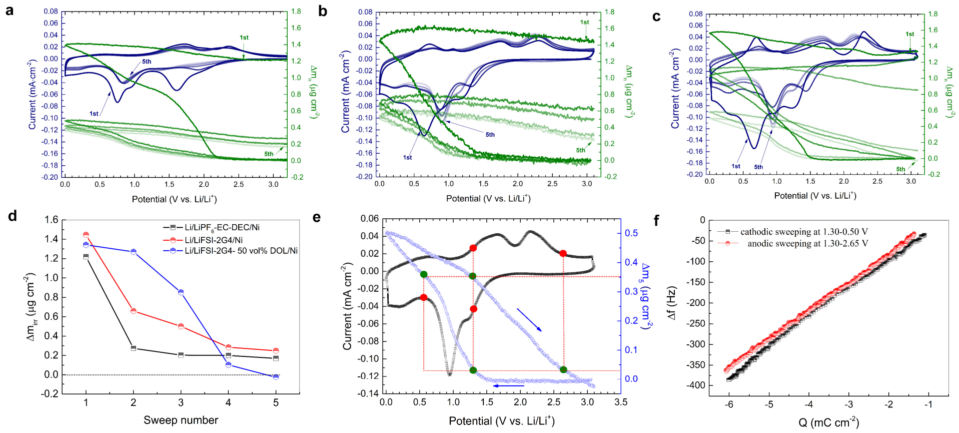

Supplementary Figure 13. Impact of CV-premodulation ahead of the bulk lithium electrodeposition. (a-c) in-situ CV/QCM study of three-electrode cells, where Ni is working electrode, using different electrolytes at 10 mV s^{-1} and ambient temperature in the range of 0-3.1 V. (a) 1 M $LiPF_6$-EC-DEC; (b) LiFSI-2G4; (c) LiFSI-2G4-50 vol% DOL. (d) variation of Δm_{irr} of Ni upon sweeping in the different electrolytes. (e) in-situ CV/QCM study on the underpotential Li electrodeposition/stripping in the 5th sweep using LiFSI-2G4-50 vol% DOL (f) Δf vs. Q plots derived from different potential ranges of the fifth-sweep using the electrolyte of LiFSI-2G4-50 vol% DOL.

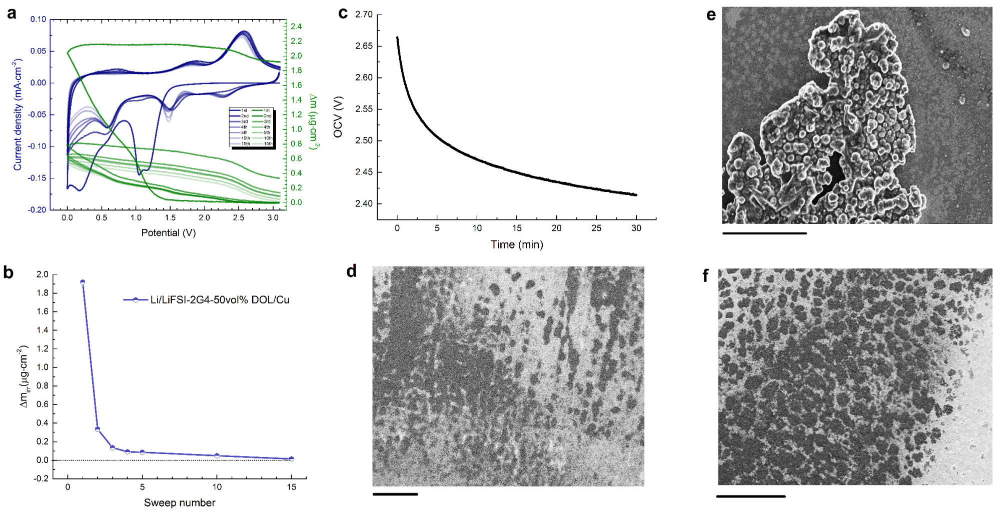

Supplementary Figure 14. Impact of CV-premodulation ahead of the bulk lithium electrodeposition. (a) in-situ CV/QCM study of three-electrode cells using Cu as the working electrode in the DSIL of LiFSI-2G4-50 vol % DOL at 10 mV s^{-1} and ambient temperature in the range of 0-3.1 V. (b) variation of Δm_{irr} of Cu upon sweeping in the DSIL of LiFSI-2G4-50 vol% DOL. (c) OCV decay of Li|Cu cells with time after twenty-sweeps with a sweep-rate of 5 mV s^{-1} in the range of 0-3.0 V. (d, e) patch film and dendrite-free particles of lithium electrodeposition observed onto Cu substrate disassembled from the cell (f) and the cell of Fig. 3 (d). Scale bar: (d)-500 µm; (e)-20 µm; (f)-500 µm.

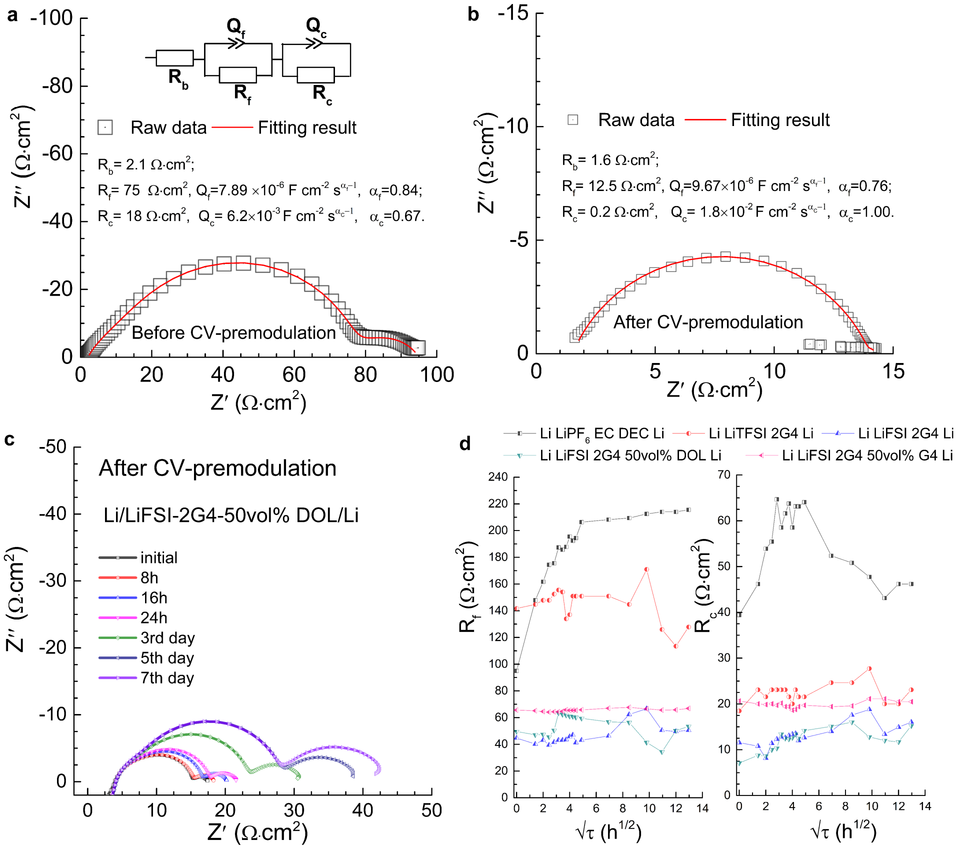

Supplementary Figure 15. Impedance spectra of symmetrical Li|Li cells. (a, b) fitting results of two typical impedance spectra using the equivalent circuit model; (c) impedance evolution of symmetrical Li|Li cell with the recovery-time after the CV-premodulation using LiFSI-2G4-50 vol% DOL; (d) R_f and R_c evolution of symmetrical Li|Li cell in the different electrolytes with the static aging-time at 25 °C.

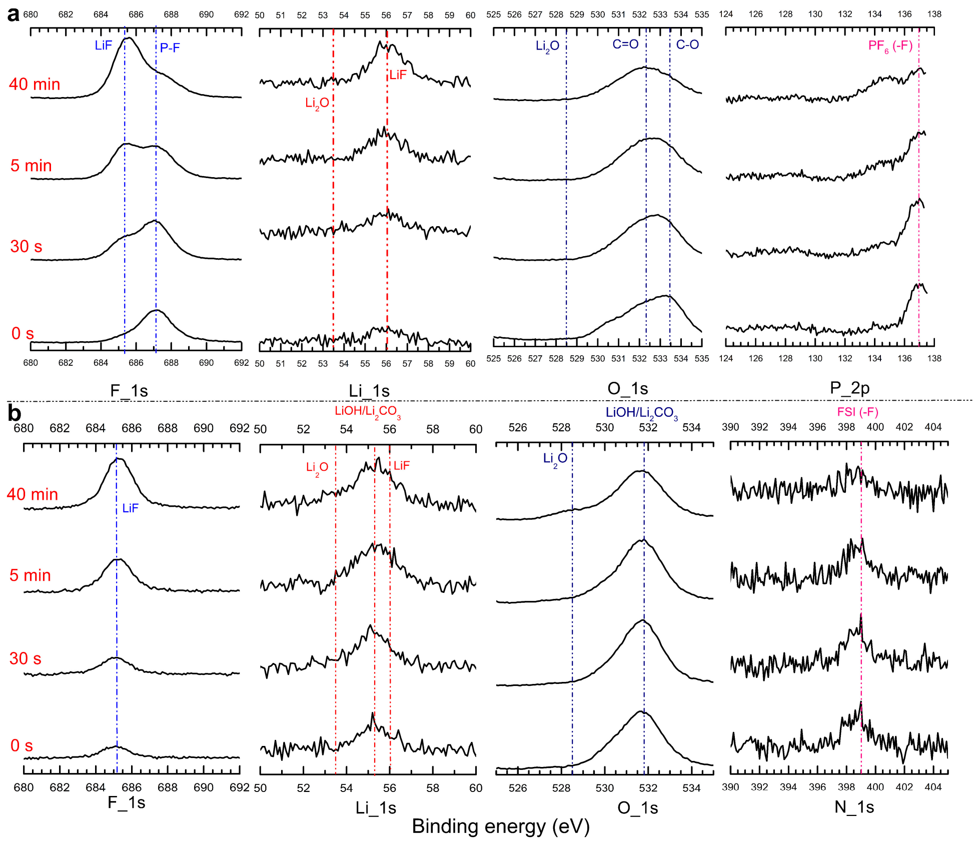

Supplementary Figure 16. Surface film chemistry of the metallic lithium electrode. (a) in 1M $LiPF_6$ in EC-DEC and (b) in LiFSI-2G4.

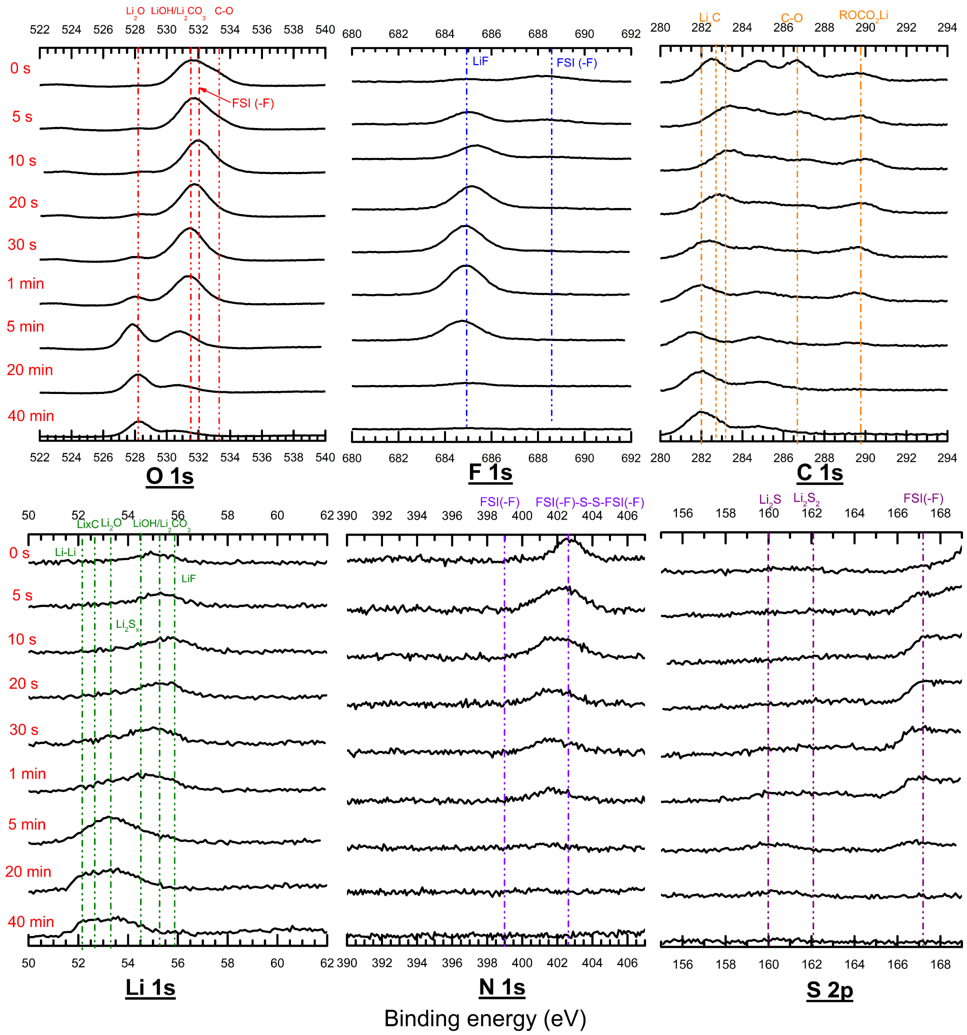

Supplementary Figure 17. Surface film chemistry of the metallic lithium electrode in contact with LiFSI-2G4-50 vol% DOL.

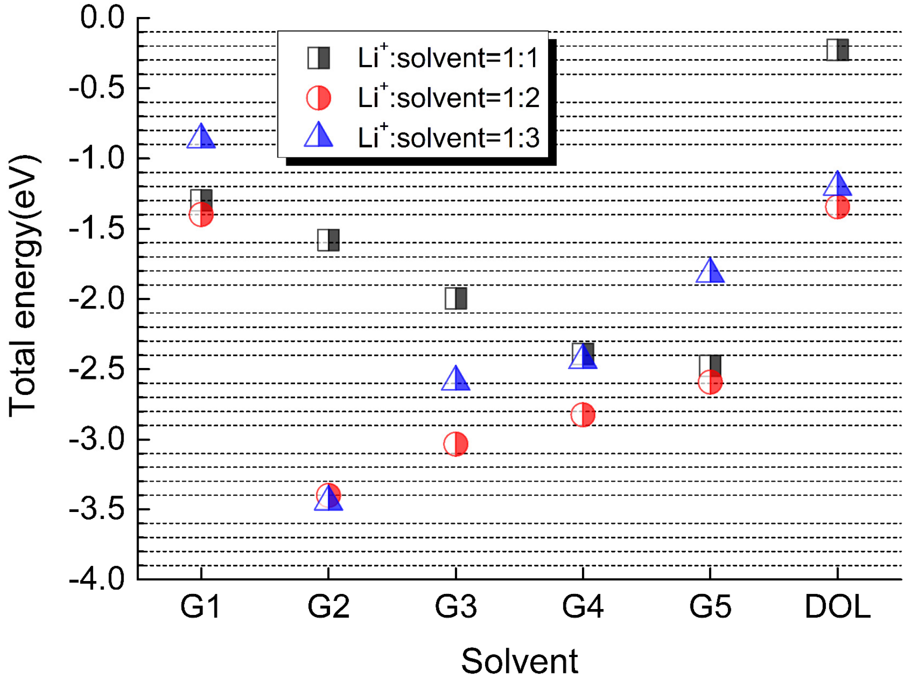

Supplementary Figure 18. Solvent-dependent total energy for the most-stable coordination structure between Li^+ and solvent in various molar ratios.

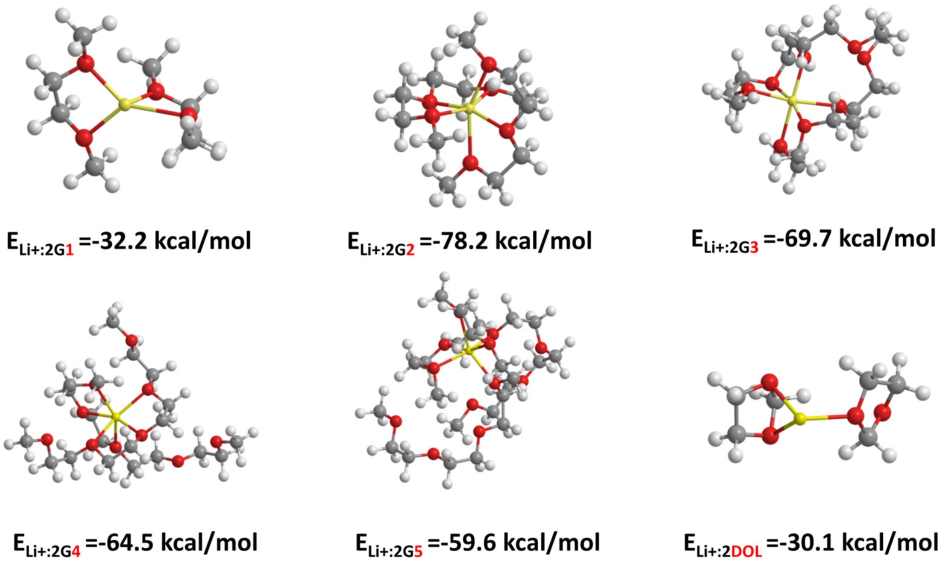

Supplementary Figure 19. Simulation results on the most-stable coordination structure between Li^+ and solvent.

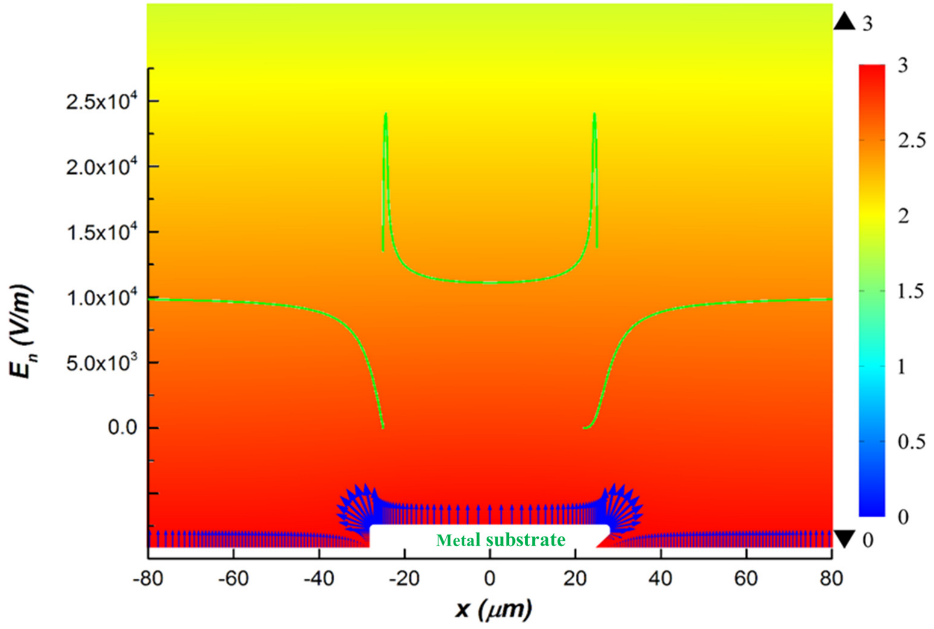

Supplementary Figure 20. The simulation of electric field distribution across planar metal patch under 3.0 V. (height: 5 μm; width: 50 μm).
